# Comparison of mortality and cause of death between adults with and without hypertrophic cardiomyopathy

**DOI:** 10.1038/s41598-022-10389-4

**Published:** 2022-04-16

**Authors:** Soonil Kwon, Hyung-Kwan Kim, Bongseong Kim, Hyun-Jung Lee, Kyung-Do Han, In-Chang Hwang, Yeonyee E. Yoon, Jun-Bean Park, Heesun Lee, Seung-Pyo Lee, Goo-Yeong Cho, Yong-Jin Kim

**Affiliations:** 1 Section of Cardiovascular Imaging, Division of Cardiology, Department of Internal Medicine and Cardiovascular Center, Seoul National University and Seoul National University Hospital, 103 Daehak-ro, Jongno-gu, Seoul, 03080 South Korea; 2 Department of Statistics and Actuarial Science, Soongsil University, Seoul, Republic of Korea; 3 Department of Cardiology, Cardiovascular Center, Seoul National University Bundang Hospital, Seongnam, Gyeonggi Republic of Korea

**Keywords:** Cardiology, Epidemiology

## Abstract

Insufficient evidence is available comparing mortality and cause of death between general hypertrophic cardiomyopathy (HCM) and general non-HCM populations. We aimed to investigate how causes of death and mortality differ in subjects with and without HCM. Using the National Health Insurance Service database from 2009 to 2016, individuals who underwent health check-up(s) with or without a history of HCM were identified. Participants in the HCM group were matched at a 1:1 ratio with those in the non-HCM group using propensity scores calculated from the baseline covariates. Mortality rates and risks were compared between the groups. In total, 14,858 participants (7,429 each in the HCM and non-HCM groups) were followed up over a mean 4.4 ± 2.2 years (mean age, 61.0 years; male proportion, 66.8%). Compared to the non-HCM group, the HCM group showed a higher risk of all-cause and HCM-related mortality and a similar risk for non-cardiovascular mortality (hazard ratio [95% confidence interval] 1.57 [1.38–1.78], 2.71 [1.92–3.83], and 1.04 [0.88–1.23], respectively). The sensitivity analyses consistently showed that the HCM group showed higher risks of all-cause and HCM-related mortality than the non-HCM group. The female participants with HCM were associated with an increasing trend of the risks of all-cause mortality but not HCM-related mortality compared to their male counterparts (p for interaction < 0.001 and 0.185, respectively). In conclusion, compared to the non-HCM population, the general HCM population showed higher risks of both all-cause and HCM-related mortality, but had a similar risk of non-cardiovascular mortality.

## Introduction

Hypertrophic cardiomyopathy (HCM) is a common inherited cardiomyopathy that can clinically manifest with sudden cardiac death, heart failure, stroke, and arrhythmia^1^. Compared to the general population, patients with HCM were traditionally recognised as having a higher cardiovascular mortality rate^2^. However, the prognosis of HCM has improved significantly because of the recent advances in disease management, risk stratification, and family screening^3^. Additionally, a previous study reported that the life expectancy of patients with HCM might not be significantly different from that of the general population^4^. Therefore, it is necessary to update how the prognosis of HCM population differs from that of the non-HCM population in this contemporary management era. Currently, only a few studies are available that directly compared mortality between HCM and non-HCM populations^5–7^. Besides, a few earlier studies included patients with HCM who had a considerable burden of comorbidities, including hypertension and heart failure^8,9^. Thus, it remains unclear whether the impact of HCM on mortality is innate or secondary to other comorbidities. In addition, previous studies mostly investigated ‘referral’ patients with HCM^5,7^, so they are inevitably susceptible to selection bias, as this population is likely to have an increased proportion of high-risk patients, making it challenging to represent the general HCM population^10^. Therefore, this study was designed to compare the cause of death and mortality between the general HCM and non-HCM populations using propensity score matching of a nationwide cohort.

## Methods

### Ethical statement and data availability

This study conforms to the ethical guidelines of the Declaration of Helsinki revised in 2013 and was approved by the institutional review board of our institution (Seoul National University Hospital Institutional Review Board, No. 1905-106-1035). The need for informed consent was waived by the Seoul National University Hospital Institutional Review Board because the study used anonymised data. All raw data are accessible from the designated terminals approved by the National Health Insurance Service (NHIS).

### Data source

This nationwide cohort study used the NHIS database of Korea. A summary of the database was previously reported^11^. In brief, the NHIS is the single public insurer that covers the entire Korean population and encourages any eligible Korean adult to receive general health check-ups provided by the NHIS biannually. Therefore, the NHIS database includes individual demographic information, history of diagnoses, and results of health check-ups. Additionally, we obtained the mortality data from Statistics Korea^12^, a government agency that provides causes of death for the entire Korean population based on the diagnostic codes on death certificates. Individuals’ history of diagnoses and causes of death are all coded according to the International Classification of Disease, Tenth Revision, Clinical Modification.

### Study design

**Figure 1:**
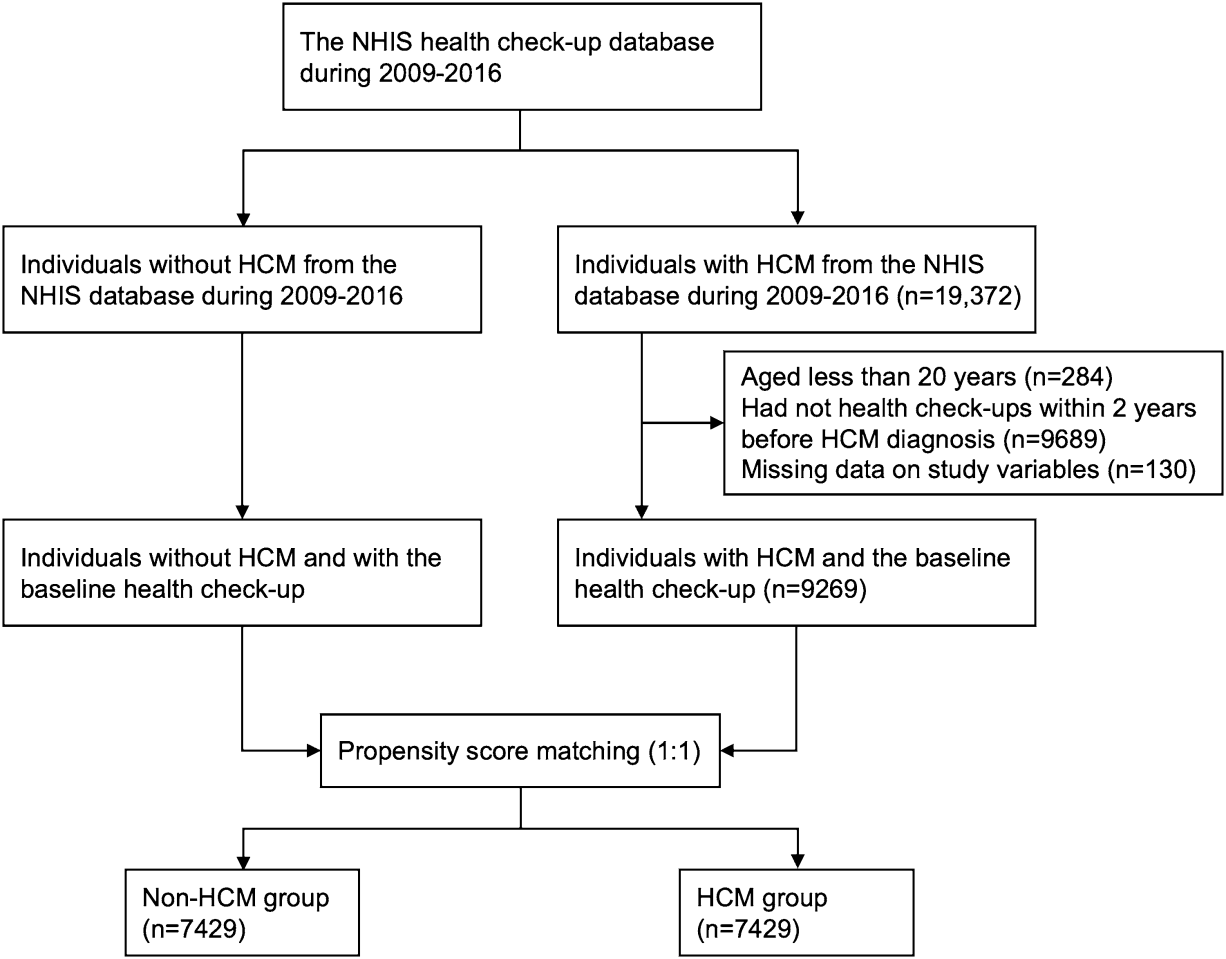
The flowchart of the study design. Using the NHIS database, subjects who were diagnosed with HCM between 2009 and 2016 were selected and exactly matched at a 1:1 ratio with the non-HCM group using a propensity score. *HCM* hypertrophic cardiomyopathy, *NHIS* National Health Insurance Service.

From 2009 to 2016, the NHIS database identified all individuals with HCM (n = 19,372) who were initial candidates for the HCM group. Individuals were excluded if they were younger than 20 years of age (n = 284), had no health check-ups within 2 years from the diagnosis of HCM (n = 9689), or had missing data for the study variables (n = 130). For the non-HCM group, we identified individuals without any history of HCM diagnosis from the same database. Then the two groups were matched at a 1:1 ratio using propensity scores calculated from the baseline covariates, resulting in 7429 participants for each group (Fig. 1). After propensity scoring matching, the two groups were well balanced (Fig. S1). Every participant was followed up from the date of the baseline health check-up until 31 December 2017. The primary outcome was set as all-cause mortality.

### Definition of hypertrophic cardiomyopathy diagnosis and causes of death

In this study, HCM was confirmed when there were both the claims for diagnostic codes I42.1 or I42.2 at either admission or outpatient clinic visit and the registration of the Rare Intractable Diseases program (RID) for HCM^12,13^. RID is a policy implemented by the Ministry of Health and Welfare to expand medical insurance for rare diseases. For this reason, accurate test results and expert opinions are required to register patients with HCM into the RID. Because third-party experts review medical data during RID registration, patients with HCM registered with RID were reliable. Causes of death were categorised into three groups: HCM-related (including arterial thromboembolism, atrial fibrillation, sudden cardiac death, cerebrovascular disease, heart failure, and ventricular arrhythmia), other cardiovascular (including hypertensive disease, ischemic heart disease, and peripheral vascular disease), and non-cardiovascular causes (including all causes of death except I-codes). Detailed information or definitions are described in more detail in Table S1.

### Study covariates

For each study participant, all study covariates were measured on the visit day for a health check-up. The covariates included age, sex, and physical measurements (height, body weight, body mass index, waist circumference, and blood pressures). We obtained information on health habits (smoking status, alcohol consumption, and regular exercise) from the survey for the health check-up. The blood test results (lipid profiles, serum creatinine level, and estimated glomerular filtration rate) were also collected. We investigated each participant’s comorbidities by reviewing a history of claimed diagnostic codes from the NHIS database. Detailed descriptions of each covariate are presented in Table S1.

### Statistical analyses

In propensity score matching, a covariate was considered balanced between the groups if its absolute standardised difference (ASD) was < 0.1. For each category of causes of death, a survival analysis was performed using the Kaplan–Meier method. Mortality rates were calculated in 1,000 person-year. A log-rank test was used to evaluate the difference in survival between the groups. Subgroup analyses were performed for age strata (< 60 and ≥ 60 years) and sex.

Two sensitivity analyses were performed to support the main results (Fig. S2). The first sensitivity analysis used the 1:1 age- (exact) and sex-matched non-HCM population. Multivariate Cox proportional hazard regression analysis was used to estimate the mortality risks, and the data are presented as adjusted hazard ratios (HRs) with 95% confidence intervals (CIs). The second sensitivity analysis was performed to investigate the mortality of participants with HCM without significant coronary artery disease (i.e. having both ischaemic heart disease [IHD] and a history of percutaneous coronary intervention [PCI]). The HCM population without significant coronary artery disease was matched at a 1:1 ratio with the non-HCM population using the propensity scores. In this case, non-HCM individuals with significant coronary artery disease were also excluded from the non-HCM group. We performed falsification analyses to evaluate whether there was a significant selection bias between the groups. Falsification outcomes included herniated intervertebral disc (M51), sinusitis (H65-67), urinary incontinence (F980, N393, N394, and R32), and cataract (H25, H26, H28, and Q120).

For all analyses, p-values < 0.05 rejected the null hypothesis. SAS version 9.3 (SAS Institute, Cary, NC, USA) was used to perform all statistical analyses.

### Declaration of Helsinki

This study conforms to the ethical guidelines of the Declaration of Helsinki revised in 2013.

## Results

In total, 14,858 participants (7,429 each in the HCM and non-HCM groups) were included in the final analysis. The distribution of the two groups’ propensity scores was equalised after the matching process (Fig. S1). Additionally, after the matching process, all covariates were well balanced between the groups without statistical difference (ASD < 0.1 for each covariate). Baseline characteristics of the study population are presented in Table 1. Participants’ mean age was 61.0 years, and the proportion of men was 66.8% (n = 9,929). The most common comorbidity was hypertension (53.5%), and the mean systolic blood pressure was 127.5 ± 15.9 mmHg. The proportions of atrial fibrillation, IHD with PCI, heart failure, and implantable cardioverter defibrillator (ICD) implantation were 7.1%, 2.0%, 11.5%, and 0.1%, respectively.

**Table 1:** Baseline characteristics of the study population after propensity score matching.

	Non-HCM group (n = 7429)	HCM group (n = 7429)	ASD
**Demographics**			
Age (year)			
Mean	61.1 ± 12.8	60.8 ± 12.3	0.019
< 60	3109 (41.8)	3401 (45.8)	
≥ 60	4320 (58.2)	4028 (54.2)	
Male	4920 (66.2)	5009 (67.4)	0.026
Smoking status			
Non-smoker	3967 (53.4)	3903 (52.5)	0.017
Ex-smoker	1795 (24.2)	1834 (24.7)	0.012
Current smoker	1667 (22.4)	1692 (22.8)	0.008
Alcohol consumption			
None	4236 (57.0)	4139 (55.7)	0.026
Mild	2641 (35.6)	2703 (36.4)	0.017
Heavy	552 (7.4)	587 (7.9)	0.018
Regular exercise	1545 (20.8)	1613 (21.7)	0.022
Low-income status	1234 (16.6)	1171 (15.8)	0.023
**Anthropometrics**			
Height (cm)	162.8 ± 9.6	163.1 ± 9.3	0.027
Body weight (kg)	66.6 ± 12.2	66.6 ± 11.7	0.005
Body mass index (kg/m^2^)	25.0 ± 3.3	24.9 ± 3.2	0.014
Waist circumference (cm)	85.4 ± 8.9	85.4 ± 8.6	0.000
SBP (mmHg)	127.7 ± 15.2	127.2 ± 16.6	0.027
DBP (mmHg)	77.7 ± 10.0	77.5 ± 11.0	0.022
**Comorbidities**			
Obesity	3712 (50.0)	3595 (48.4)	0.032
Hypertension	4051 (54.5)	3940 (53.0)	0.030
Diabetes mellitus	1194 (16.1)	1104 (14.9)	0.034
Dyslipidemia	3004 (40.4)	2826 (38.0)	0.049
Atrial fibrillation	489 (6.6)	563 (7.6)	0.039
IHD with PCI	151 (2.0)	151 (2.0)	0
Heart failure	797 (10.7)	905 (12.2)	0.046
Peripheral arterial disease	1167 (15.7)	991 (13.3)	0.067
Ischemic stroke	593 (8.0)	564 (7.6)	0.015
Renal disease	527 (7.1)	441 (5.9)	0.047
Cancer	363 (4.9)	372 (5.0)	0.006
ICD implantation	8 (0.1)	10 (0.1)	0.008
**Laboratory tests**			
Total cholesterol (mg/dL)	192.0 ± 40.3	191.3 ± 38.7	0.019
Triglyceride (mg/dL)	126.6 (125.1–128.2)	124.2 (122.7–125.7)	0.037
HDL (mg/dL)	51.6 ± 20.7	52.0 ± 19.7	0.018
LDL (mg/dL)	112.7 ± 43.6	112.2 ± 43.1	0.014
Serum creatinine (mg/dL)	1.0 ± 1.1	1.1 ± 1.0	0.037
eGFR (mL/min/1.73 m^2^)	83.3 ± 33.5	81.9 ± 48.5	0.034

Data are n (%) or mean ± standard deviation except for triglyceride (median with interquartile range).

*HCM* hypertrophic cardiomyopathy, *ASD* absolute standardized difference, *SBP* systolic blood pressure, *DBP* diastolic blood pressure, *IHD* ischemic heart disease, *PCI* percutaneous coronary intervention, *ICD* implantable cardioverter-defibrillator, *HDL* high-density lipoprotein, *LDL* low-density lipoprotein, *eGFR* estimated glomerular filtration rate.

### Mortality according to cause of death

Over a mean follow-up of 4.4 ± 2.2 years, 1,011 (6.8%) cases of all-cause mortality were observed. Compared to the non-HCM group, the HCM group had significantly higher all-cause (8.3% versus 5.3%, p < 0.001), HCM-related (1.6% versus 0.6%, p < 0.001), and other cardiovascular mortalities (0.8% versus 0.5%, p = 0.006) (Table 2). Non-cardiovascular mortality was not significantly different between the two groups (p = 0.600). Regarding major causes of cardiovascular-related mortality, the HCM group had significantly higher mortality due to cerebrovascular disease (0.9% versus 0.3%, p < 0.001), IHD (0.7% versus 0.4%, p = 0.032), heart failure (0.3% versus 0.1%, p = 0.034), and atrial fibrillation (0.2% versus < 0.1%, p < 0.001) than the non-HCM group (Table 2). Mortality due to sudden cardiac death and ventricular arrhythmia was not significantly different between the two groups (p = 0.133 and 0.655, respectively), possibly due to low incidences of both events.

**Table 2:** Mortality according to cause of death.

	Non-HCM group (n = 7429)	HCM group (n = 7429)	p
**Cause of death**			
All-cause	392 (5.3)	619 (8.3)	< 0.001
HCM-related causes^a^	44 (0.6)	120 (1.6)	< 0.001
Other cardiovascular causes^b^	35 (0.5)	62 (0.8)	0.006
Non-cardiovascular causes	265 (3.6)	277 (3.7)	0.600
**Major causes of cardiovascular-related death**			
Cerebrovascular disease	25 (0.3)	69 (0.9)	< 0.001
Ischemic heart disease	30 (0.4)	49 (0.7)	0.032
Heart failure	10 (0.1)	22 (0.3)	0.034
Atrial fibrillation	1 (< 0.1)	14 (0.2)	< 0.001
Sudden cardiac death	5 (0.1)	11 (0.2)	0.133
Ventricular arrhythmia	3 (< 0.1)	2 (< 0.1)	0.655

Data are n (%).

*HCM* hypertrophic cardiomyopathy.

^a^Including atrial fibrillation, cerebrovascular disease, heart failure, sudden cardiac death, arterial thromboembolism, and ventricular arrhythmia.

^b^Including hypertensive disease, ischemic heart disease, and peripheral arterial disease.

### Mortality rates and risks according to cause of death

**Figure 2:**
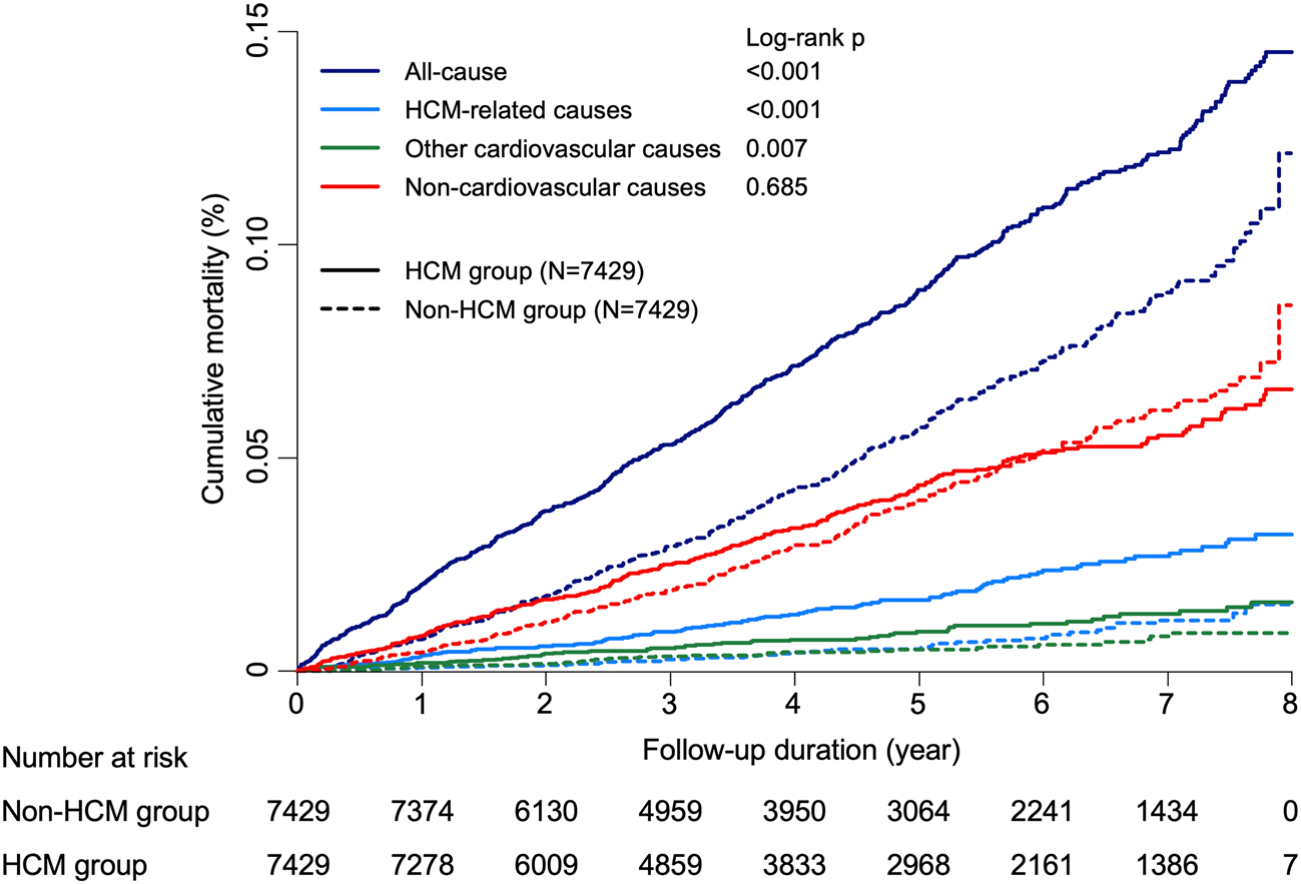
Cumulative mortality according to the cause of death. All-cause mortality, HCM-related mortality, and other cardiovascular mortality were significantly higher in the HCM group compared with those in the non-HCM group. Mortality from non-cardiovascular causes, however, were not significantly different between the HCM and the non-HCM groups. *HCM* hypertrophic cardiomyopathy.

**Figure 3:**
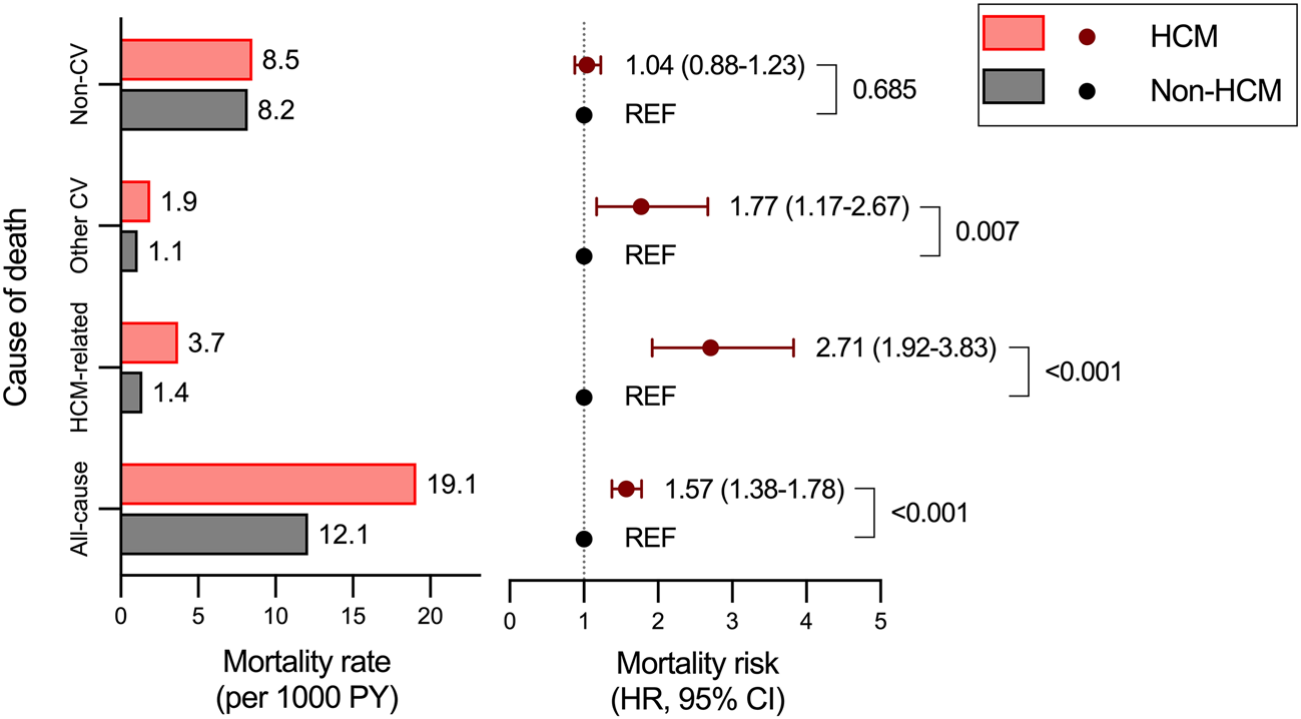
Mortality rates and risks according to the cause of death. Mortality rates, mortality risks with 95% CIs, and p-values are presented. Mortality rates were higher in the HCM group than in the non-HCM group, irrespective of the cause of death. Compared to the non-HCM group, the HCM group was associated with significantly higher risks for all-cause, HCM-related, and other cardiovascular mortality. Non-cardiovascular mortality was comparable in the HCM group versus the non-HCM group. *HCM* hypertrophic cardiomyopathy, *CV* cardiovascular, *REF* reference, *PY* person-year, *HR* hazard ratio, *CI* confidence interval.

The cumulative incidence of mortality was significantly different between the two groups except for non-cardiovascular causes (Fig. 2). Compared to the non-HCM group, the HCM group had significantly higher mortality rates due to all-cause (19.1 versus 12.1 per 1,000 person-years, p < 0.001), HCM-related causes (3.7 versus 1.4 per 1,000 person-years, p < 0.001), and other cardiovascular causes (1.9 versus 1.1 per 1,000 person-years, p < 0.007), and had no difference in non-cardiovascular causes (8.5 versus 8.2 per 1,000 person-years, p = 0.609) (Fig. 3**)**. Also, the HCM group was significantly associated with higher risks of mortality except for non-cardiovascular mortality than those of the non-HCM group (HR [95% CI] 1.57 [1.38–1.78], 2.71 [1.92–3.83], 1.77 [1.17–2.67], and 1.04 [0.88–1.23] for all-cause, HCM-related, other cardiovascular, and non-cardiovascular mortality, respectively) (Fig. 3). Mortality rates and risks by specific disease categories are presented in Table S2. Among the disease categories, the HCM group was significantly associated with increased risks of (in the highest order of) atrial fibrillation, ischaemic stroke, cerebrovascular disease (including ischaemic stroke), heart failure, and IHD compared to the non-HCM group (HR [95% CI] 14.2 [1.87–108.04], 3.26 [1.60–6.62], 2.74 [1.73–4.33], 2.15 [1.02–4.54], and 1.64 [1.04–2.58], respectively). Mortality risks of other disease categories, including sudden cardiac death and ventricular arrhythmia, were not significantly different between the two groups.

### Subgroup, sensitivity, and falsification analyses

**Figure 4:**
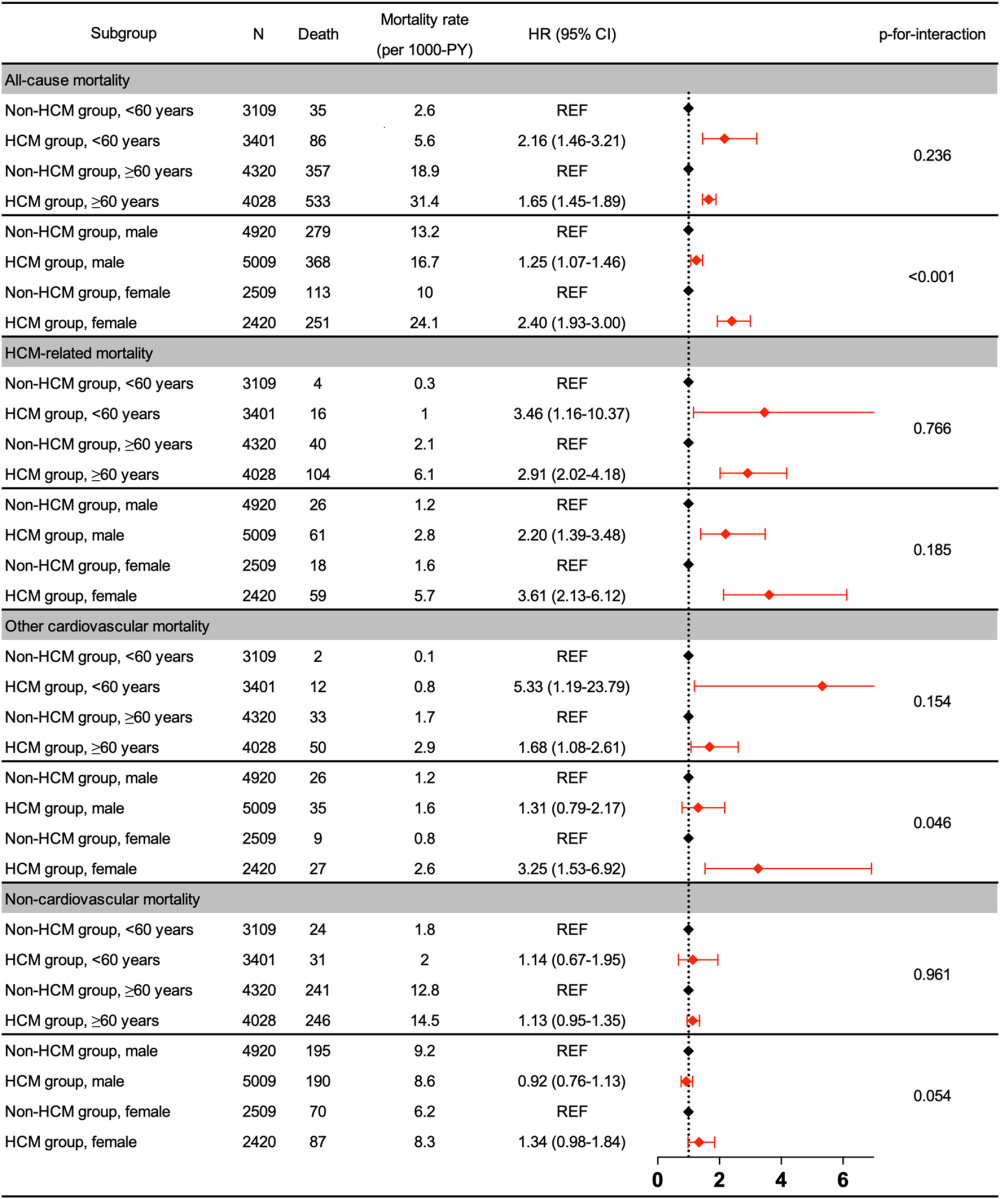
Subgroup analyses. *PY* person-year, *HR* hazard ratio, *CI* confidence interval, *HCM* hypertrophic cardiomyopathy, *REF* reference.

Participants with HCM aged both < 60 and ≥ 60 years were associated with increased risks of all-cause, HCM-related causes, and other cardiovascular mortalities with the exception of non-cardiovascular mortality compared to their non-HCM counterparts (all-cause mortality: HR [95% CI] 2.16 [1.46–3.21] and 1.65 [1.45–1.89]; HCM-related mortality: 3.46 [1.16–10.37] and 2.91 [2.02–4.18]; other cardiovascular mortality: 5.33 [1.19–23.79] and 1.68 [1.08–2.61]; and non-cardiovascular mortality: 1.14 [0.67–1.95] and 1.13 [0.95–1.35], respectively). However, there were no significant interactions between the two groups for the subgroups of age strata (< 60 and ≥ 60 years) (Fig. 4).

We observed that female participants with HCM had a trend of higher risks of all-cause and other cardiovascular mortalities than male participants with HCM (all-cause mortality: HR [95% CI] 2.40 [1.93–3.00] versus 1.25 [1.07–1.46]; other cardiovascular mortality: 3.25 [1.53–6.92] versus 1.31 [0.79–2.17]), when the non-HCM counterparts served as the reference (p for interaction < 0.001 and 0.046, respectively). However, no significant difference was noted in the HCM-related mortality between male and female participants with HCM (HR [95% CI] 2.20 [1.39–3.48] versus 3.61 [2.13–6.12], p for interaction = 0.185) (Fig. 4).

Results of the sensitivity analyses using multivariate Cox regression analysis are presented in Tables S3 and S4. Compared to the age- and sex-matched non-HCM group, the HCM group had a higher burden of comorbidity (Table S3). The multivariate Cox regression analysis reproduced the primary results except for other cardiovascular mortality, which showed no significant difference between the two groups (Table S4). The sensitivity analysis excluding participants with significant coronary artery disease also reproduced the primary results except for other cardiovascular mortality (Table S5). The HCM group showed a marginal significance of an increased risk of PCI compared to the non-HCM group (HR [95% CI] 1.24 [1.00–1.55], p = 0.053) (Table S5). There were no significant differences in the risks of falsified outcomes between the two groups (Table S6).

## Discussion

In the present study, after matching the two groups using propensity scores, we observed that the HCM group showed higher risks of all-cause, HCM-related, and other cardiovascular mortalities, but had a similar risk of non-cardiovascular mortality, compared to the non-HCM group. The current study has a few strengths. First, we investigated a nationwide cohort of adults with HCM, allowing for inclusion of both referral and non-referral individuals simultaneously. Second, we compared the HCM population to the non-HCM population matched by propensity scores calculated from 30 covariates. Third, we analysed the differences in mortality rates and risks between the two groups according to cause of death. Finally, sensitivity and falsification analyses were performed to support the main results.

Here, we observed that the most common mode of death in the HCM group was non-cardiovascular causes (Table 2), with cancer being the most common non-cardiovascular cause (Table S2). This result may be driven by extended longevity of individuals with HCM resulting from improved medical care^1^, and thus, more individuals with HCM should have more chances to be diagnosed with cancer than before. Among the major causes of cardiovascular-related death, the most common was cerebrovascular disease, followed by IHD, heart failure, and atrial fibrillation. Sudden cardiac death and ventricular arrhythmia accounted for only a minor portion of mortality. These findings appear to be different from those of previous studies^5,14^. Songsirisuk et al. reported the cause of death in ‘referral’ patients with HCM recruited from a single tertiary hospital in Thai^14^. Although this study investigated a small number of patients (n = 161), the HCM-related mortality rate was 2%/year. The common causes of death were heart failure, sudden cardiac death, and stroke. In particular, the incidence of sudden cardiac death was 1%/year, which was in clear contrast to our study finding (0.03%/year, Table S2). A possible explanation for the difference may be related to the characteristics of patients recruited; that is, the earlier study recruited referral patients from a single tertiary institution, possibly leading to a referral bias and a higher rate of sudden cardiac death due to a higher proportion of high-risk patients with HCM compared to our study. Lorenzini et al. compared 4893 patients with HCM to the general non-HCM population in Europe, and reported that patients with HCM carried a higher risk of all-cause death than the general population^5^. They found that sudden cardiac death tended to decrease with age, but it occurred in 3.4% of patients enrolled. The proportion of sudden cardiac death in our study (0.2%) was lower than that of the previous report, which can possibly be explained by the difference in the mean age of the study population (60.8 years in the present study versus 49.2 years in the earlier report)^5^. One earlier study also reported a lower incidence of sudden cardiac death events in two tertiary centre registries^15^, corroborating our study finding. The difference is again partly explained by the fact that Lorenzini et al.'s study also targeted ‘referral’ HCM patients, as in Songsirisuk et al.'s report^14^, while we targeted general individuals with HCM from a nationwide cohort^5^. Besides, phenotypic differences between Eastern and Western countries may partly modify the prognosis of HCM, although the HCM phenotype cannot be differentiated in our nationwide HCM cohort.

We observed that females with HCM had a higher risk of all-cause mortality than their male counterparts, as observed in previous studies^5,16,17^. Additionally, we found that other cardiovascular mortality was higher in women than in men, while there was no significant difference in HCM-related mortality between the sexes. A previous report also found that there was no difference in HCM-related mortality between the sexes owing to the advances in contemporary management^18^, although females with IHD are at a higher risk of mortality than their male counterparts^19^. Especially, post-menopausal women are associated with a higher risk of hypertension and its subsequent cardiovascular complications than men due to the loss of vasodilatory effects of endogenous oestrogen Regarding non-cardiovascular mortality, female participants with HCM had only a marginal significance of increased risks compared to their male counterparts (p for interaction = 0.054) (Fig. 4). Taken together, these results suggest that female subjects with HCM may be more vulnerable to other cardiovascular mortality that was composed of hypertensive disorders, IHD, and peripheral arterial disease, than their male counterparts.

The Cox regression analysis for the sensitivity analysis showed that there was no difference in other cardiovascular and non-cardiovascular mortalities between the HCM and non-HCM groups (Table S4). In another sensitivity analysis that was performed after excluding participants with IHD and PCI, we again found no difference in other cardiovascular and non-cardiovascular mortalities between the two groups (Table S5). Therefore, the general participants with HCM still mainly suffer from HCM-related causes of mortality even in the contemporary management era; therefore, health education regarding HCM is required for the general population. More active education, early detection/diagnosis, and close follow-up may be helpful to reduce HCM-related causes of mortality in the general HCM population. This issue needs to be re-evaluated in the future.

Of interest, we observed that the HCM group had a marginal significance of a higher risk of PCI compared to the non-HCM group (Table S5). This phenomenon cannot be explained by the differences in cardiovascular risk factors, such as obesity, hypertension, diabetes mellitus, and dyslipidaemia, because the HCM and non-HCM groups were carefully matched using propensity scores. The most plausible explanation is that the individuals with HCM may be more regularly and closely monitored in a dedicated centre, which may facilitate detection and treatment of significant coronary disease. Thanks to the improved contemporary management strategy, the longevity of individuals with HCM has been significantly extended^1^. Nevertheless, individuals with HCM intentionally reduce physical activity at work and leisure time after being diagnosed with HCM because of a concern for sudden cardiac death^20^. However, individuals with HCM are not immune from cardiovascular diseases, such as coronary disease. Given that elderly individuals with HCM have a relatively low risk of sudden cardiac death, maintaining good cardiovascular fitness through appropriate physical activity may be beneficial and improve prognosis^12^. However, only recently have individuals with HCM been advised to perform regular exercise at an appropriate level^21^. In this respect, the risk of coronary artery disease requiring PCI observed in the current study should be focused on, especially in elderly individuals with HCM.

## Limitations

A few study limitations should be acknowledged. First, the validity of the HCM diagnosis needs to be checked. Defining the HCM population by using diagnostic codes may have falsely estimated the actual HCM population. However, we used both the diagnostic codes and RID codes to improve the diagnostic accuracy. Such a definition validated a reasonable diagnostic accuracy (sensitivity, 91.5%; specificity, 100%; and positive predictive value, 92.6%) in our previous study^13^. In addition, the NHIS database has been widely and reliably used for HCM research^8,12,22–26^. Second, this study cannot be completely free from selection bias of the study population. To investigate the baseline characteristics at the time of HCM diagnosis, we screened the individuals who had a health check-up within 2 years from the diagnosis. However, this process may have preferentially selected individuals who are more interested in their health status, leading to a reduction in the mortality rate. Third, the generalisation of our results to the younger HCM population is limited because our study analysed the HCM population with a mean age of 61 years. Fourth, the follow-up periods of the study population were relatively short (4.4 ± 2.2 years). Fifth, although our study used a representative HCM population in South Korea, it was impossible to differentiate patients who were treated in the HCM specialty center from those who were not. This information may give further insights on the special need of HCM specialty center. Sixth, the study results might be partly influenced by survivorship bias. We excluded the HCM population aged less than 20 years, and the mean age of the study population is about 61 years. Thus, the resultant study population could be survivors from sudden cardiac death or those with a low risk for sudden cardiac death. This may explain a low sudden cardiac death rate observed in our study. However, Asian HCM population was reported to have a lower sudden cardiac death rate, even for the referral patients to the HCM specialty center^15^. Also, our study includes both referred and non-referred populations, and non-referred HCM patients were reported to have lower mortality^10^. Seventh, this study did not comprehensively investigate drug uses, and the difference in the uses of beta-blockers or calcium channel blockers may convey a bias in the study results. Eighth, the database we used in the current study do not allow for evaluating genetic testing results or echocardiography-based left ventricular outflow tract obstruction. Finally, it is difficult to infer any causality related to our results because the study was retrospective in nature.

## Conclusions

This nationwide cohort study compared mortality and cause of death between the general HCM and general non-HCM matched populations. Among the major causes of cardiovascular-related mortality, cerebrovascular disease, IHD, heart failure, and atrial fibrillation were significantly more frequent in the HCM population than in the non-HCM population. Multiple statistical analyses consistently showed that compared to the non-HCM population, the HCM population had significantly higher risks of all-cause and HCM-related mortality. Our results suggest that such an increased mortality risk be an innate feature of HCM, independent of other concomitant comorbidities. Appropriate HCM-specialised medical care and surveillance may aid in further improvement in the prognosis of individuals with HCM in the modern era.

## Supplementary Information

Supplementary Information.

## Data Availability

The data is available from the Korean National Health Insurance Sharing Service (NHISS; https://nhiss.nhis.or.kr/) database which is open to researchers on request with approval by the Insitutional Review Board.
