# Supplementary material for: Comparison of mortality and cause of death between adults with and without hypertrophic cardiomyopathy
Source: Sci Rep. 2022 Apr 16;12:6386. doi: 10.1038/s41598-022-10389-4 (PMC9013352; doi:10.1038/s41598-022-10389-4)
Supplement: Supplementary file 1 — Supplementary Information. [file 41598_2022_10389_MOESM1_ESM.docx]

Supplementary Appendix to Manuscript Entitled

Comparison of Mortality and Cause of Death between Adults with and without Hypertrophic Cardiomyopathy

Table of Contents

Table S1. Definitions of covariates and causes of death2

Table S2. Mortality rates and risks by specific disease categories 4

Table S3. Comparison of baseline characteristics of the HCM group and the age- and sex-matched non-HCM group 6

Table S4. Sensitivity analysis: multivariate Cox proportional hazard regression analysis 8

Table S5. Sensitivity analysis: exclusion of the population with significant IHD 9

Table S6. Falsification analysis 10

Figure S1. Distributions of propensity scores of the study population: before and after the matching 11

Figure S2. The flowchart of the sensitivity analyses 12

**Table S1. Definitions of covariates and causes of death**

| Covariates defined by the results of health check-ups | | |
| --- | --- | --- |
| Covariates | Definition | |
| Smoking status | Categorised into non-, ex- and current smokers, based on the answers of the questionnaire provided by the NHIS | |
| Alcohol consumption | Categorised into none, mild (<30 g/day) and heavy drinkers (≥30 g/day), based on the answers of the questionnaire provided by the NHIS | |
| Regular exercise | Moderate physical activity more than 30 minutes ≥5 days/week or strenuous physical activity more than 20 minutes ≥3 days/week, based on the answers of the questionnaire provided by the NHIS | |
| Low-income status | Indicated if a respondent's answer on the annual income belonged to the lower 10 percentile of the income distribution of all Korean population | |
| Covariates defined by operational definitions using diagnostic codes | | |
| Covariates | ICD-10-CM code | Operational definition |
| Atrial fibrillation | I48 | Admission ≥1 or outpatient clinic ≥2 |
| Cancer | C00-97 | Admission ≥1 or outpatient clinic ≥2 |
| Diabetes mellitus | E11-E14; and minimum 1 prescription of anti-diabetic drugs (sulfonylureas, metformin, meglitinides, thiazolidinediones, dipeptidyl peptidase-4 inhibitors, α-glucosidase inhibitors, SGLT2-inhibitors, GLP-1 agonists, and insulin). | Admission ≥1 or outpatient department ≥2 |
| Dyslipidemia | E78 | Admission ≥1 or outpatient clinic ≥2 Minimum 1 prescription of lipid-lowering medication (statin, ezetimibe, fenofibrate) |
| Heart failure | I50, I42.0, I11.0, I13.0, I13.2 | Admission ≥ 1 or outpatient clinic ≥2 |
| Hypertension | I10-I13, I15; and minimum 1 prescription of anti-hypertensive drug (thiazide, loop diuretics, aldosterone antagonist, alpha-/beta-blocker, calcium-channel blocker, angiotensin-converting enzyme inhibitor, angiotensin II receptor blocker). | Admission ≥1 or outpatient department ≥2 |
| ICD implantation | Diagnostic codes: None  Procedure codes: O0211, O0212  Device codes: G8301, G8302, G8303 | Both procedure and device codes;  ICD implantation - O0211 + G8301 or 8302 (single- or dual-chamber); ICD replacement - O0212 + G8301 or 8302; CRT-D - O0211 + G8303 |
| IHD with PCI | Diagnostic codes: I20-25  Procedure codes: M6551, M6552, M6561-6564, M6571, and M6572 | Both diagnostic and procedural codes during admission ≥1 |
| Ischemic stroke | I63, I64 | Admission ≥1 or outpatient clinic ≥2 |
| Peripheral arterial disease | I70.2, I73 | Admission ≥1 or outpatient clinic ≥2 |
| Renal disease | N00-007, N11, I12, N18-19, Q61 | Admission ≥1 or outpatient clinic ≥2 |
| Causes of death | ICD-10-CM code | Operational definition |
| HCM-related causes | arterial thromboembolism (I74), atrial fibrillation (I48), cardiac arrest (I46), cerebrovascular diseases (I60-69), heart failure (I50), and ventricular arrhythmia (I49) | |
| Other cardiovascular causes | hypertensive diseases (I10-15), ischemic heart diseases (I20-25), and peripheral vascular diseases (I70, I73) | |
| Non-cardiovascular causes | All causes of death except I-codes | |

Abbreviations: NHIS, National Health Insurance Service; ICD-10-CM, International Classification of Disease, Tenth Revision, Clinical Modification; SGLT2, sodium-glucose cotransporter-2; GLP-1, glucagon-like peptide-1; ICD, implantable cardioverter-defibrillator; CRT, cardiac resynchronization therapy; IHD, ischemic heart disease; PCI, percutaneous coronary intervention; HCM, hypertrophic cardiomyopathy.

**Table S2. Mortality rates and risks by specific disease categories**

|  | N | Death | Follow-up duration  (per 1000-PY) | Mortality rate  (per 1000-PY) | HR (95% CI) | p |
| --- | --- | --- | --- | --- | --- | --- |
| Atrial fibrillation (I48) |  |  |  |  |  |  |
| Non-HCM group | 7429 | 1 | 32,419 | <0.1 | Reference | 0.010 |
| HCM group | 7429 | 14 | 32,431 | 0.4 | 14.2 (1.87-108.04) |  |
| Cancer (C-code) |  |  |  |  |  |  |
| Non-HCM group | 7429 | 124 | 32,419 | 3.8 | Reference | 0.092 |
| HCM group | 7429 | 153 | 32,431 | 4.7 | 1.23 (0.97-1.56) |  |
| Cerebrovascular disease (I60-69) |  |  |  |  |  |  |
| Non-HCM group | 7429 | 25 | 32,419 | 0.8 | Reference | <0.001 |
| HCM group | 7429 | 69 | 32,431 | 2.1 | 2.74 (1.73-4.33) |  |
| Diabetes mellitus (E10-14) |  |  |  |  |  |  |
| Non-HCM group | 7429 | 13 | 32,419 | 0.4 | Reference | 0.831 |
| HCM group | 7429 | 12 | 32,431 | 0.4 | 0.92 (0.42-2.01) |  |
| Digestive disease (K-code) |  |  |  |  |  |  |
| Non-HCM group | 7429 | 17 | 32,419 | 0.5 | Reference | 0.812 |
| HCM group | 7429 | 17 | 32,431 | 0.5 | 0.92 (0.47-1.82) |  |
| Endocrinology disease (E-code) |  |  |  |  |  |  |
| Non-HCM group | 7429 | 14 | 32,419 | 0.4 | Reference | 0.485 |
| HCM group | 7429 | 18 | 32,431 | 0.6 | 1.28 (0.64-2.58) |  |
| Heart failure (I50) |  |  |  |  |  |  |
| Non-HCM group | 7429 | 10 | 32,419 | 0.3 | Reference | 0.045 |
| HCM group | 7429 | 22 | 32,431 | 0.7 | 2.15 (1.02-4.54) |  |
| Hypertension (I10-15) |  |  |  |  |  |  |
| Non-HCM group | 7429 | 5 | 32,419 | 0.2 | Reference | 0.077 |
| HCM group | 7429 | 13 | 32,431 | 0.4 | 2.54 (0.90-7.14) |  |
| Infectious disease (A-code, B-code) |  |  |  |  |  |  |
| Non-HCM group | 7429 | 15 | 32,419 | 0.5 | Reference | 0.731 |
| HCM group | 7429 | 13 | 32,431 | 0.4 | 0.88 (0.42-1.85) |  |
| Ischemic heart disease (I20-25) |  |  |  |  |  |  |
| Non-HCM group | 7429 | 30 | 32,419 | 0.9 | Reference | 0.034 |
| HCM group | 7429 | 49 | 32,431 | 1.5 | 1.64 (1.04-2.58) |  |
| Ischemic stroke (I63-64) |  |  |  |  |  |  |
| Non-HCM group | 7429 | 10 | 32,419 | 0.3 | Reference | 0.001 |
| HCM group | 7429 | 33 | 32,431 | 1.0 | 3.26 (1.60-6.62) |  |
| Respiratory disease (J-code) |  |  |  |  |  |  |
| Non-HCM group | 7429 | 41 | 32,419 | 1.3 | Reference | 0.795 |
| HCM group | 7429 | 44 | 32,431 | 1.4 | 1.06 (0.69-1.62) |  |
| Sudden cardiac death (I46) |  |  |  |  |  |  |
| Non-HCM group | 7429 | 5 | 32,419 | 0.2 | Reference | 0.138 |
| HCM group | 7429 | 11 | 32,431 | 0.3 | 2.22 (0.77-6.40) |  |
| Trauma (S-code, T-code) |  |  |  |  |  |  |
| Non-HCM group | 7429 | 44 | 32,419 | 1.4 | Reference | 0.115 |
| HCM group | 7429 | 30 | 32,431 | 0.9 | 0.69 (0.43-1.10) |  |
| Ventricular arrhythmia (I49) |  |  |  |  |  |  |
| Non-HCM group | 7429 | 3 | 32,419 | 0.1 | Reference | 0.666 |
| HCM group | 7429 | 2 | 32,431 | 0.1 | 0.67 (0.11-4.04) |  |

Abbreviations: PY, person-year; HR, hazard ratio; CI, confidence interval; HCM, hypertrophic cardiomyopathy.

**Table S3. Comparison of baseline characteristics of the HCM group and age- and sex-matched non-HCM group**

|  | Non-HCM group  (n=8348) | HCM group  (n=8348) | p |
| --- | --- | --- | --- |
| Demographics |  |  |  |
| Age (year) |  |  | 1 |
| Mean | 61.0±11.4 | 61.0±11.4 |  |
| <60 | 3713 | 3713 |  |
| ≥60 | 4635 | 4635 |  |
| Male | 5705 (68.3) | 5705 (68.3) | 1 |
| Smoking status |  |  | 0.010 |
| Non-smoker | 4386 (52.5) | 4306 (51.6) |  |
| Ex-smoker | 1975 (23.7) | 2140 (25.6) |  |
| Current smoker | 1987 (23.8) | 1902 (22.8) |  |
| Alcohol consumption |  |  | 0.634 |
| None | 4695 (56.2) | 4646 (55.7) |  |
| Mild | 2972 (35.6) | 3031 (36.3) |  |
| Heavy | 681 (8.16) | 671 (8.0) |  |
| Regular exercise | 1815 (21.7) | 1797 (21.5) | 0.735 |
| Low income status | 1431 (17.1) | 1305 (15.6) | 0.008 |
| Anthropometrics |  |  |  |
| Height (cm) | 163.2±9.4 | 163.3±9.2 | 0.392 |
| Body weight (kg) | 64.8±11.5 | 67.2±11.6 | <0.001 |
| Body mass index (kg/m^2^) | 24.3±3.2 | 25.1±3.2 | <0.001 |
| Waist circumference (cm) | 83.6±8.5 | 85.9±8.6 | <0.001 |
| SBP (mmHg) | 126.4±15.6 | 127.1±16.7 | 0.005 |
| DBP (mmHg) | 77.8±10.3 | 77.5±11.0 | 0.143 |
| Comorbidities |  |  |  |
| Obesity | 3272 (39.2) | 4252 (50.9) | <0.001 |
| Hypertension | 3398 (40.7) | 4700 (56.3) | <0.001 |
| Diabetes mellitus | 1297 (15.5) | 1243 (14.9) | 0.245 |
| Dyslipidemia | 2188 (26.2) | 3461 (41.6) | <0.001 |
| Atrial fibrillation | 371 (4.4) | 1168 (14.0) | <0.001 |
| IHD with PCI | 32 (0.4) | 169 (2.0) | <0.001 |
| Heart failure | 622 (7.5) | 1511 (18.1) | <0.001 |
| Peripheral arterial disease | 808 (9.7) | 1027 (12.3) | <0.001 |
| Ischemic stroke | 341 (4.1) | 692 (8.3) | <0.001 |
| Renal disease | 302 (3.6) | 491 (5.9) | <0.001 |
| Cancer | 281 (3.4) | 395 (4.7) | <0.001 |
| ICD implantation | 6 (0.1) | 68 (0.8) | <0.001 |
| Laboratory tests |  |  |  |
| Total cholesterol (mg/dL) | 194.6±39.5 | 190.2±39.0 | <0.001 |
| Triglyceride (mg/dL) | 4.8±0.5 | 4.8±0.5 | 0.066 |
| HDL (mg/dL) | 53.0±20.2 | 51.7±21.0 | <0.001 |
| LDL (mg/dL) | 115.1±48.1 | 112.1±50.4 | <0.001 |
| Serum creatinine (mg/dL) | 1.00±0.81 | 1.1±1.3 | <0.001 |
| eGFR (mL/min/1.73m^2^) | 86.6±51.2 | 81.2±45.0 | <0.001 |

Data are n (%) or mean ± standard deviation.

Abbreviations: HCM, hypertrophic cardiomyopathy; SBP, systolic blood pressure; DBP, diastolic blood pressure; IHD, ischemic heart disease; PCI, percutaneous coronary intervention; ICD, implantable cardioverter-defibrillator; HDL, high-density lipoprotein; LDL, low-density lipoprotein; eGFR, estimated glomerular filtration rate.

**Table S4. Sensitivity analysis: multivariate Cox proportional hazard regression analysis**

|  | N | Death | Mortality rate  (per 1000-PY) | Model 1  HR (95% CI) | Model 2  Adjusted HR (95% CI) | Model 3  Adjusted HR (95% CI) |
| --- | --- | --- | --- | --- | --- | --- |
| All-cause death |  |  |  | p <0.001 | p <0.001 | p =0.017 |
| Non-HCM group | 8348 | 495 | 13.8 | Reference | Reference | Reference |
| HCM group | 8348 | 680 | 19.2 | 1.40 (1.24-1.57) | 1.41 (1.25-1.58) | 1.16 (1.03-1.31) |
| HCM-related causes^1^ |  |  |  | p <0.001 | p <0.001 | p <0.001 |
| Non-HCM group | 8348 | 54 | 1.5 | Reference | Reference | Reference |
| HCM group | 8348 | 145 | 4.1 | 2.73 (1.99-3.73) | 2.77 (2.02-3.78) | 1.96 (1.41-2.71) |
| Other cardiovascular causes^2^ |  |  |  | p =0.167 | p =0.150 | p =0.925 |
| Non-HCM group | 8348 | 51 | 1.4 | Reference | Reference | Reference |
| HCM group | 8348 | 65 | 1.8 | 1.30 (0.90-1.87) | 1.31 (0.91-1.89) | 0.98 (0.67-1.44) |
| Non-cardiovascular causes |  |  |  | p =0.397 | p =0.364 | p =0.869 |
| Non-HCM group | 8348 | 286 | 8.0 | Reference | Reference | Reference |
| HCM group | 8348 | 302 | 8.5 | 1.07 (0.91-1.26) | 1.08 (0.92-1.27) | 1.01 (0.86-1.20) |

1 Including atrial fibrillation, cerebrovascular disease, heart failure, sudden cardiac death, arterial thromboembolism, and ventricular arrhythmia.

2 Including hypertension, ischemic heart disease, and peripheral arterial disease.

Model 1: non-adjusted.

Model 2: adjusted with age and sex.

Model 3: adjusted with age, sex, low-income status, smoking status, alcohol consumption, regular exercise, and comorbidities.

Abbreviations: PY, person-year; HR, hazard ratio; CI, confidence interval; HCM, hypertrophic cardiomyopathy.

**Table S5. Sensitivity analysis after excluding patients with significant coronary artery disease^1^**

|  | N | Death | Follow-up duration  (per 1000-PY) | Mortality rate  (per 1000-PY) | HR (95% CI) | p |
| --- | --- | --- | --- | --- | --- | --- |
| All-cause death |  |  |  |  |  |  |
| Non-HCM group | 7245 | 369 | 31,839 | 11.6 | Reference | <0.001 |
| HCM group | 7245 | 590 | 31,832 | 18.5 | 1.560 (1.40-1.82) |  |
| HCM-related causes^2^ |  |  |  |  |  |  |
| Non-HCM group | 7245 | 47 | 31,839 | 1.5 | Reference | <0.001 |
| HCM group | 7245 | 114 | 31,832 | 3.6 | 2.39 (1.70-3.36) |  |
| Other cardiovascular causes^3^ |  |  |  |  |  |  |
| Non-HCM group | 7245 | 41 | 31,839 | 1.3 | Reference | 0.153 |
| HCM group | 7245 | 55 | 31,832 | 1.7 | 1.34 (0.90-2.01) |  |
| Non-cardiovascular causes |  |  |  |  |  |  |
| Non-HCM group | 7245 | 239 | 31,839 | 7.5 | Reference | 0.139 |
| HCM group | 7245 | 272 | 31,832 | 8.5 | 1.14 (0.96-1.36) |  |
| Percutaneous coronary intervention |  |  |  |  |  |  |
| Non-HCM group | 7245 | 143 | 31,480 | 4.5 | Reference | 0.053 |
| HCM group | 7245 | 177 | 31,290 | 5.7 | 1.24 (1.00-1.55) |  |

1 defined as the patients with ischemic heart disease and a history of percutaneous coronary intervention.

2 Including atrial fibrillation, cerebrovascular disease, heart failure, sudden cardiac death, arterial thromboembolism, and ventricular arrhythmia.

3 Including hypertension, ischemic heart disease, and peripheral arterial disease.

Abbreviations: PCI, percutaneous coronary intervention; PY, person-year; HR, hazard ratio; CI, confidence interval; HCM, hypertrophic cardiomyopathy.

**Table S6. Falsification analysis**

|  | N | Death | Follow-up duration  (per 1000-PY) | Incidence rate  (per 1000-PY) | HR (95% CI) | p |
| --- | --- | --- | --- | --- | --- | --- |
| Herniated intervertebral disc |  |  |  |  |  |  |
| Non-HCM group | 7429 | 462 | 31,385 | 14.7 | Reference | 0.501 |
| HCM group | 7429 | 485 | 31,466 | 15.4 | 1.02 (0.90-1.16) |  |
| Sinusitis |  |  |  |  |  |  |
| Non-HCM group | 7429 | 1172 | 29,359 | 39.9 | Reference | 0.548 |
| HCM group | 7429 | 1208 | 29,467 | 41.0 | 1.02 (0.94-1.11) |  |
| Urinary incontinence |  |  |  |  |  |  |
| Non-HCM group | 7429 | 2249 | 26,334 | 85.4 | Reference | 0.850 |
| HCM group | 7429 | 2260 | 26,381 | 85.7 | 0.99 (0.93-1.05) |  |
| Cataract |  |  |  |  |  |  |
| Non-HCM group | 7429 | 2469 | 25,519 | 96.8 | Reference | 0.414 |
| HCM group | 7429 | 2517 | 25,578 | 98.4 | 1.01 (0.96-1.07) |  |

Herniated intervertebral disc: diagnostic code of M51.

Sinusitis: diagnostic codes of H65, H66, and H67.

Urinary incontinence: diagnostic codes of F980, N393, N394, and R32.

Cataract: diagnostic codes of H25, H26, H28, and Q120.

Abbreviations: PY, person-year; HR, hazard ratio; CI, confidence interval; HCM, hypertrophic cardiomyopathy.

**Figure S1. Distributions of propensity scores of the study population: before and after the matching**


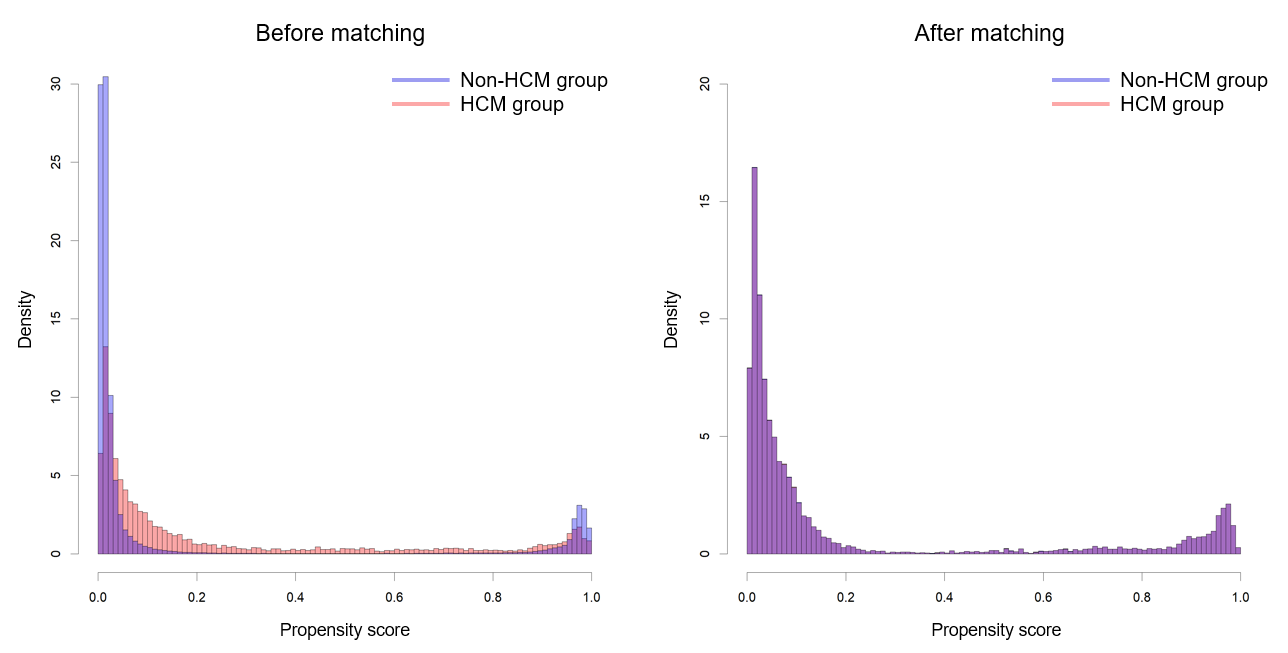


Abbreviation: HCM, hypertrophic cardiomyopathy group.

**Figure S2. The flowchart of the sensitivity analyses**


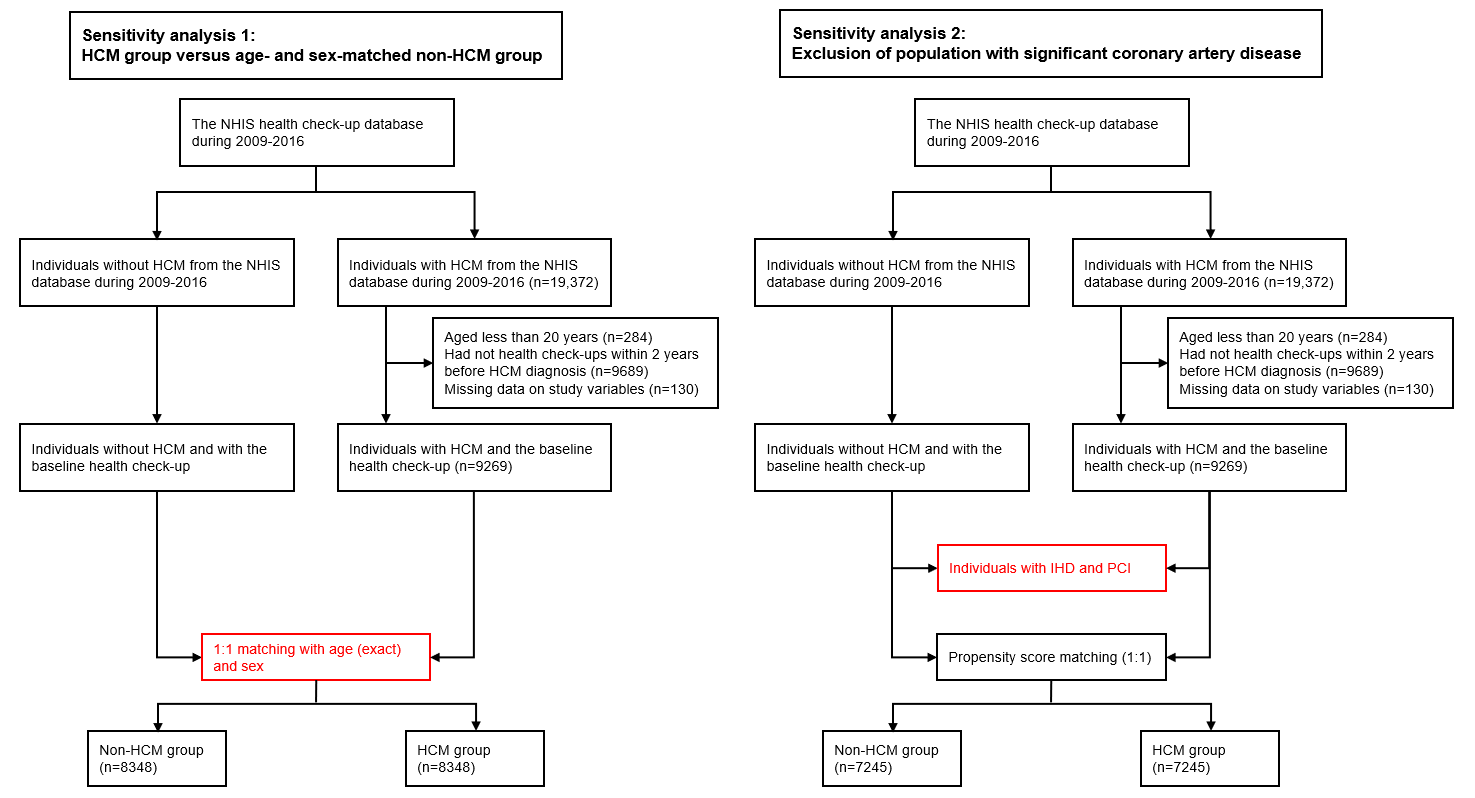


Abbreviations: HCM, hypertrophic cardiomyopathy; NHIS, National Health Insurance Service; IHD, ischemic heart disease; PCI, percutaneous coronary intervention.
